# An Adaptive Early Stopping Technique for DenseNet169-Based Knee Osteoarthritis Detection Model

**DOI:** 10.3390/diagnostics13111903

**Published:** 2023-05-29

**Authors:** Bander Ali Saleh Al-rimy, Faisal Saeed, Mohammed Al-Sarem, Abdullah M. Albarrak, Sultan Noman Qasem

**Affiliations:** 1 Department of Computer Science, Faculty of Computing, Universiti Teknologi Malaysia, Johor Bahru 81310, Malaysia; 2 DAAI Research Group, Department of Computing and Data Science, School of Computing and Digital Technology, Birmingham City University, Birmingham B4 7XG, UK; faisal.saeed@bcu.ac.uk; 3 College of Computer Science and Engineering, Taibah University, Medina 41477, Saudi Arabia; msarem@taibahu.edu.sa; 4 Computer Science Department, College of Computer and Information Sciences, Imam Mohammad Ibn Saud Islamic University (IMSIU), Riyadh 11432, Saudi Arabia; amsbarrak@imamu.edu.sa (A.M.A.); snmohammed@imamu.edu.sa (S.N.Q.)

**Keywords:** knee OA detection, DenseNet169, early stopping, self-adaptive, GCE

## Abstract

Knee osteoarthritis (OA) detection is an important area of research in health informatics that aims to improve the accuracy of diagnosing this debilitating condition. In this paper, we investigate the ability of DenseNet169, a deep convolutional neural network architecture, for knee osteoarthritis detection using X-ray images. We focus on the use of the DenseNet169 architecture and propose an adaptive early stopping technique that utilizes gradual cross-entropy loss estimation. The proposed approach allows for the efficient selection of the optimal number of training epochs, thus preventing overfitting. To achieve the goal of this study, the adaptive early stopping mechanism that observes the validation accuracy as a threshold was designed. Then, the gradual cross-entropy (GCE) loss estimation technique was developed and integrated to the epoch training mechanism. Both adaptive early stopping and GCE were incorporated into the DenseNet169 for the OA detection model. The performance of the model was measured using several metrics including accuracy, precision, and recall. The obtained results were compared with those obtained from the existing works. The comparison shows that the proposed model outperformed the existing solutions in terms of accuracy, precision, recall, and loss performance, which indicates that the adaptive early stopping coupled with GCE improved the ability of DenseNet169 to accurately detect knee OA.

## 1. Introduction

Osteoarthritis is a degenerative joint disorder that affects millions of people worldwide, leading to pain and loss of mobility in the affected joints [1,2]. Early detection and diagnosis of osteoarthritis are crucial for effective treatment, but traditional diagnostic methods can be time-consuming and invasive. In recent years, deep learning-based techniques have shown great potential in the early detection of knee osteoarthritis using medical imaging [2,3]. This type of approach can automate the analysis of radiographic images, reducing the dependence on subjective interpretations, and increasing the accuracy and consistency of diagnosis [4,5].

Deep learning is a powerful tool used for image analysis, pattern recognition, and decision making. It is based on the use of artificial neural networks, which are modelled after the human brain, and can learn from data [6]. In the context of knee osteoarthritis detection, deep learning algorithms can be trained to recognize patterns and features that are indicative of the disease in radiographic images [7,8]. The ability to automatically extract and analyze these features can provide a more objective and accurate diagnosis than traditional methods [8,9]. Recent studies have shown that deep learning-based techniques can achieve high accuracy in the detection of knee osteoarthritis using X-ray and MRI images [9,10]. These results demonstrate the potential of deep learning-based techniques for the early detection of knee osteoarthritis and pave the way for the development of more accurate and efficient diagnostic tools.

However, it is worth noting that knee osteoarthritis detection using deep learning is still in the development phase, and more research is required to validate and improve these solutions before they become widely adopted in clinical practice [11]. One of the main challenges in deep learning-based solutions for osteoarthritis detection is overfitting, which occurs when a model is trained on a limited dataset and performs well on the training data but performs poorly on new, unseen data [12,13]. According to [14], the limited size and diversity of available datasets can lead to poor generalization and overfitting of the models. This can result in a high accuracy on the training set but a low accuracy on the test set and real-world data [15].

Increasing the number of epochs in a deep learning model can also lead to overfitting and affect the accuracy of the model in various ways [16]. Generally speaking, as the number of training epochs increases, the model will continue to learn from the training data, and the accuracy of the training set will typically increase [17]. However, as the model continues to learn, it may start to overfit the training data, resulting in a decrease in the accuracy on the test set or real-world data [18].

In the case of a DenseNet169 model, which is a type of convolutional neural network, increasing the number of epochs can lead to an improvement in the accuracy on the training set. The model will be able to learn more from the training data, and the weights and biases of the network will be adjusted to better fit the data. However, after a certain number of epochs, the accuracy on the validation set may start to decrease, indicating that the model has started to overfit [19].

It is worth noting that the optimal number of epochs will depend on the specific data, the architecture of the model, and the task at hand [20]. One way to determine the optimal number of epochs is to use techniques such as early stopping, which involves monitoring the performance of the model on a validation set and stopping the training when the performance starts to degrade [21]. By stopping the training before the model starts to overfit, early stopping can help to prevent overfitting and improve the accuracy of the model on new, unseen data.

Recent research has proposed various early stopping methods to improve the accuracy of DenseNet169-based models. For example, Ref. [21] proposed an early stopping strategy that monitors the performance of the model on the validation set and stops the training when the performance starts to degrade. In addition, Refs. [22,23] employed the early stopping regularization that monitors the performance of the model on the validation set and stops the training when the performance starts to degrade. In their study, Ref. [13] investigated knee OA early detection, and OA grading identification using deep learning. The researchers developed a new approach to classify data in deep learning models using the Laplace distribution-based strategy (LD-S) and created an aggregated multiscale dilated convolution network (AMD-CNN) to extract features from multivariate data of knee osteoarthritis (KOA) patients. They combined the AMD-CNN and LD-S to create a new KOA-CAD method that achieves three objectives in computer-aided diagnosis. Similarly, Ref. [24] introduced a new method for identifying knee osteoarthritis (KOA) in its early stages, which involves using deep learning to extract features from data and classify it. The algorithm being suggested utilizes X-ray images to both train and test the results. It uses hybrid feature descriptors, which extract features through combinations of CNN with HOG and CNN with LBP. The system employs three multi-classifiers to categorize diseases based on the KL grading system using KNN, RF, and SVM. However, there are several current research issues related to early stopping methods in deep learning models including the DenseNet169. One issue is determining the optimal stopping point [23]. The optimal stopping point will depend on the specific data, the architecture of the model, and the task at hand, and it is not clear yet how to determine it in an automated and general way that works well across different datasets and tasks [25,26]. Another issue is related to the trade-off between the accuracy and the generalization of the model. While early stopping can prevent overfitting, it can also result in underfitting if the model is stopped too early; this can lead to decreased accuracy on the test set or real-world data [27]. Therefore, there is a need to strike a balance between preventing overfitting and ensuring that the model has enough capacity to generalize well to new data. Additionally, the definition of performance degradation can vary depending on the dataset and the task, which makes it difficult to generalize early stopping methods across different datasets and tasks [28].

To this end, this study is devoted to investigating the applicability and efficacy of a novel adaptive early stopping technique in DenseNet169 in the context of knee OA detection. Our early stopping mechanism sets a patience threshold for early stopping by calculating the running average of the validation loss. In such a way, our technique can avoid arbitrary termination of the training. The proposed technique also embeds a novel gradual cross-entropy coefficient for accurate loss estimation during the early stopping of model training. The contribution of this paper is three-fold, as follows:

1. An adaptive early stopping technique was proposed for DenseNet169 that dynamically adjusts the number of epochs and the batch size during the training, based on the contribution of each batch to the accuracy of the model.
2. A gradual loss estimation method based on cross-entropy was proposed for measuring the dissimilarity between the predicted class probabilities and the true class labels.
3. An improved DenseNet169-based knee OA detection model which incorporates the techniques in (1) and (2) was developed and experimentally evaluated using the Knee Osteoarthritis Severity Grading dataset.

The rest of this paper is structured as follows. Section 2 provides the details on the methodology design and techniques proposed in this study. Section 3 describes the dataset and experimental environment used to carry out the model evaluation. It also explains the results and analytically discusses the findings from the experimental evaluation. Section 4 concludes the paper with suggestions for further research suggestions.

### Related Works

The evolution in detecting and assessing the severity level of knee OA has seen a transition from traditional methods to the utilization of advanced machine learning and deep learning techniques. These include the use of complex network theory [11], circular Fourier filters [2], and deep learning algorithms to analyze radiographic knee X-ray images and aid in the early detection and diagnosis of the disease. A pivotal development in this research is the usage of a deep learning-based algorithm to automatically assess and grade the OA severity, often achieving comparable accuracy with expert radiologists [7,8]. In some instances, utilizing deep learning techniques on properly preprocessed images, such as through image sharpening, has resulted in improved accuracy rates [24]. Similarly, a semi-automatic model based on deep Siamese convolutional neural networks has been used to detect OA lesions according to the KL scale [29]. Furthermore, transfer learning has been deployed to aid the classification performance of models trained on imbalanced datasets.

With the advancement in deep learning architectures, new methodologies for OA severity assessment are introduced. A variety of deep-learning models have been proposed in the literature for diagnosing the severity of knee OA. For example, Ref. [30] leveraged a fully convolutional network (FCN) to locate knee joints and a deep convolutional neural network (CNN) to differentiate various stages of knee OA severity. Likewise, Ref. [31] introduced a technique using deep Siamese CNNs for automatic grading of knee OA severity following the KL grading scale, treating knee OA as a multi-class problem based on KL grades. Moreover, Ref. [32] presented a Discriminative Regularized Auto-Encoder (DRAE) for the early detection of knee OA, specifically differentiating between non-OA and minimal OA. The DRAE combines a discriminative loss function with the standard auto-encoder training criterion to improve the identification of knee OA.

Pre-trained deep learning models such as DenseNet and ResNet were also used in several studies for the knee OA severity level assessment. In particular, DenseNet201 was employed in [33] to develop knee OA grading. The model trains the DenseNet201 architecture on knee radiographic images from the OAI dataset. Using the Kellgren and Lawrence (KL) grading system, the model classifies the severity from grade 0 through grade 4. Similarly, the knee OA model proposed in [34] utilized DenseNet169. The model involves training the DenseNet169 using a balanced combination of two loss functions, categorical cross-entropy and mean squared error. This model inherently enables the prediction of knee OA severity on both an ordinal scale (0, 1, 2, 3, 4) and a continuous scale (0–4).

The study [35] proposed two novel learning structures, Deep Hybrid Learning-I (DHL-I) and Deep Hybrid Learning-II (DHL-II), both devised for efficient knee osteoarthritis (OA) severity classification based on Kellgren-Lawrence (KL) grades. DHL-I, based on a convolutional neural network (CNN), introduces a five-class prediction structure. This model is trained on knee X-ray images, then extracts features, applies principal component analysis (PCA) for dimensionality reduction, and then uses support vector machines (SVMs) for classification. DHL-II follows the same process, but the pre-trained CNN developed for DHL-I is fine-tuned using the concept of transfer learning to classify knee OA into four, three, and two classes.

When training a deep neural network model for assessing knee OA severity level, setting the appropriate number of training epochs and batch size per epoch often poses a challenge [36]. Overfitting might occur if too many epochs are used, while underfitting may result from too few epochs [37]. Training a neural network involves finding the right balance to avoid overfitting the training data. While adjusting the number of training epochs can help, it is computationally intensive and is not guaranteed to find an optimal value. Early stopping offers a more efficient solution [36]. This strategy involves training the model for many epochs, then halting the training when the model’s performance on a validation dataset starts to decline, ensuring optimal generalization performance [37]. This can be achieved by setting a potentially large number of training epochs initially, and then halting the training process when there is no further improvement in the model’s performance on the validation dataset.

Several studies have adopted early stopping for knee OA severity level assessment. A convolutional neural network with ResU-Net architecture (ResU-Net-18) was used in [38] to develop a Multiple-JSW for knee OA severity and progression. The model segments the knee X-ray images, and the minimum and multiple joint space widths (JSW) were estimated from this segmentation and verified against radiologist measurements. During ResU-Net-18 training, the early stopping mechanism was implemented. This technique ends the training if there is no reduction in the loss for 10 consecutive epochs, serving as a preventive measure against overfitting.

The study conducted by [39] developed a fully automated deep-learning model for assessing the severity of knee osteoarthritis (OA) using the Kellgren–Lawrence (KL) grading system. The algorithm was developed to use posterior–anterior (PA) and lateral (LAT) views of knee radiographs for this assessment. Early stopping was employed to halt the training before the model overfitted. The early stopping parameter was set to 20, which stops the training after 20 epochs. Nonetheless, identifying the ideal number of epochs poses a significant challenge. Similar to methods not employing early stopping, this approach could lead to overfitting if the early stopping criteria are set too high, and to underfitting if the criteria are set too low.

A pre-trained CNN model was also used in [29], which developed a semi-automatic computer-aided diagnosis (CAD) for detecting knee OA based on ResNet-34. The model used deep Siamese convolutional neural networks and a fine-tuned ResNet-34 to detect OA lesions in both knees based on the Kellgren and Lawrence (KL) scale. In order to balance the prevention of overfitting with maintaining model accuracy, an early stopping criterion was implemented. This stopped training when there was not any improvement in the validation accuracy observed after 50 epochs.

Although some of the existing knee OA grading models employ early stopping, they rely on statically set patience parameter values based on the number of epochs and the batch size. Such a static approach makes the model rigid and unable to adapt to the varying characteristics of the OA in X-ray images. If the patience value is set statically, it may not be optimal, as a value that is too high may lead to overfitting. This is because the model could continue training beyond the point of optimal generalization, learning the noise in the training data. On the other hand, a value that is too low may stop the training prematurely, leading to an underfit model that does not capture the underlying patterns in the data. Hence, it is crucial to find a balance and possibly employ dynamic strategies in setting the patience parameter for early stopping.

## 2. Materials and Methods

The methodology section of this paper describes the methods and procedures used to develop and evaluate the deep learning-based knee osteoarthritis (OA) detection method using X-ray images. We first present the dataset used in this study and the pre-processing steps applied to the images. Next, we describe the DenseNet169 architecture, and the fine-tuning process used to adapt the model to the knee OA detection task. We also describe the implementation details of the early stopping methods used to prevent overfitting and improve the accuracy of the model. Finally, we present the evaluation metrics and statistical analysis used to assess the performance of the proposed method. This section provides a detailed description of the steps taken to achieve the results and conclusions of this study, allowing for replication and further research on the topic.

### 2.1. X-ray Images Pre-Processing

The model development was carried out in three phases, i.e., pre-processing, training, and fine-tuning. At pre-processing phase, X-ray images underwent several procedures, namely, embedding, data augmentation, transposition, and flipping. Furthermore, vision transformer (ViT) was used to divide the input images into fixed-size patches and then positionally embed them into the transformer’s encoder (TE). This step reduces the overhead on the model as it replaces the convolutions while maintaining a high level of accuracy. Concretely, the ViT takes an image, x∈R(H×W×C), as input and turns it into a sequence of patches $,x_{p}\in R(N\times P\times P\times C)$, where (H,W) denotes the hight and width of the original image, C denotes the number of channels, (P,P) denotes the patch resolution, and *N* is the number of patches, which is calculated as follows.

(1) $$N=\frac{WH}{P^{2}}$$

Then, the generated patches are embedded linearly into the TE. The TE uses a multi-head self-attention layer to control the embedding and generates a richer representation of image data. In particular, the self-attention layer consolidates the ability of TE to relate the sequence of inputs with each other.

### 2.2. An Adaptive Early Stopping for DenseNet169-Based Knee OA Detection Model

**Figure 1:**
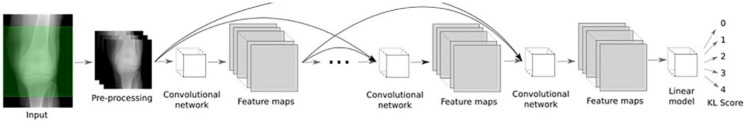
The architecture of DenseNet169.

The model adopts the DenseNet169 architecture in which each layer is connected to every other layer [40,41]. The rationale behind this choice is that DenseNet169 architecture has far fewer trainable parameters compared to other architectures. Therefore, DenseNet169 helps to increase the depth of deep CNNs while avoiding information vanishing, which happens when the path between input and output layers becomes too big. By reducing the number of parameters, DenseNet169 gets rid of redundant feature maps, which, in turn, reduces the number of filters as well [42]. Figure 1 shows the architecture of DenseNet169.

The DenseNet169 architecture is composed of several types of layers including convolutional, maxpool, dense, and transition layers [41]. Moreover, the architecture uses two activation functions, namely, Relu and SoftMax. The former is used throughout the architecture, except for the final layer, in which SoftMax is used instead. The purpose of convolutional layers is to apply multiple filters to the X-ray image and generate a feature map that describes the intensity of the extracted features. Concretely, if we take an image input with L × N size followed by a convolutional layer and apply an m × m filter, the output of the convolution will be an (l − m + 1) × (l − n + 1).

The maxpool layer in DenseNet169 is then used to decrease the feature map size. To achieve that, a pooling filter is applied over the feature map, which aggregates the features in the area covered by the filter region. Concretely, a feature map with (*n_h_*, *n_w_*, *n_c_*) dimensions can be reduced by applying the MaxPool technique as described in Equation 1 as follows:

(2) $$MaxPool=\frac{n_{c}\times (n_{h}-f+1)\times (n_{w}-f+1)}{s^{2}}$$

where *h* denotes the height, *w* denotes the width, *c* denotes the channel of the feature map, and *f* denotes the size of the filter.

The dense layer in DenseNet169 architecture consists of nodes (neurons) that receive inputs from all nodes in the preceding layer. Those inputs undergo matrix–vector multiplication. Concretely, it is assumed that *M* is an *x* × *y* matrix, *p* is a (1 × *y*) vector, and the matrix *λ* of parameters of the preceding layer was learned using the backpropagation. Therefore, the weights ($\varphi^{l_{y}}$) and biases ($\eta^{l_{y}}$) associated with layer *l_y_* can be calculated as follows:

(3) $$\varphi^{l_{y}}=\varphi^{l_{y}}-\alpha \times d\varphi^{l_{y}}$$

(4) $$\eta^{l_{y}}=\eta^{l_{y}}-\alpha \times d\eta^{l_{y}}$$

The $d\varphi^{l_{y}}$ and $d\eta^{l_{y}}$ are the partial derivatives of the loss function of *φ* and *η*. Finally, the transition layer decreases the model complexity by reducing the number of channels using 1 × 1 convolution. Table 1 shows the layered architecture of DenseNet169. It details the information for each layer, including the kernel size, tensor size, and used parameters. From the table, we can observe that Relu and SoftMax are used as activation functions. Moreover, the stride value (which determines the number of pixels that shift over the input matrix) was set to 2 in all convolutions, pooling, and transition layers. In addition, the dropout that helps in preventing overfitting and reduces the variance is set to 0.2 for all dense layers. We can also observe that the tensor size decreases by half when moving toward the output layer.

### 2.3. An Adaptive Early Stopping Technique

Unlike classical sequential models, our model dynamically adjusts the number of epochs as well as the number of steps per epoch (batch size) during the training, based on the contribution of each batch to the accuracy of the model. An early stopping mechanism was incorporated into the feedforward and backpropagation during the model training. This mechanism solves the issue of identifying an appropriate number of training epochs as well as batch size per epoch. The early stopping allows the model to start with arbitrary values for both parameters and stops the training when no further improvement happens at both levels. During the model’s training, the early stopping mechanism monitors one or more performance measures based on which the training can be aborted. In our study, we monitor the loss on the validation set. The model stops the training when no further decrement is achieved in the validation loss. To avoid immature early stopping, we set a patience threshold as a baseline value calculated using the running average of the loss difference (ε). Equation (5) was used to calculate the value of the patience parameter. The equations show that the value is updated at every step within the epoch based on the average of previous values, which avoids arbitrary stopping.

(5) $$Patience=\frac{avg(\varepsilon_{t_{i-1}})+\varepsilon_{t_{i}}}{i+1}$$

(6) $$\varepsilon =t_{i}-t_{i-1}$$

where $t_{i}$ denotes the *i*th value of the observed measure.

The model waits until the threshold’s value is satisfied, then triggers the early stopping. Such a controlling mechanism relies on two parameters, a global parameter (macro controller), and a local parameter (micro controller). On the one hand, the macro controlling parameter aborts the training when a set of preceding and current epochs make no improvement to the accuracy. On the other hand, the micro-controlling parameter aborts the running epoch at the time when it detects that no further improvement to the accuracy is made during that epoch. Therefore, model training takes less time and uses fewer resources.

However, dropping part of the data on the macro and micro level could deprive the model of valuable data located that would have been used in later epochs. To mitigate such drawback of early stopping, an improved loss function technique with the ability to compensate for potentially lost data was developed.

### 2.4. A Gradual Cross-Entropy Loss Estimation Technique

As pointed out above, the existing loss function techniques rely on the entire data allocated for the epoch to calculate the loss. However, the early stopping aborts the epoch execution and drops a portion of training data. Consequently, the loss estimation is negatively affected. To address the effect of early stopping on the accuracy of the loss estimation, our study proposes a gradual cross-sntropy (GCE) technique which improves the loss estimation at the micro (epoch) level. Unlike the existing loss calculation techniques that consider all epoch data, the GCE calculates the loss based on only the portion of the data that was consumed in the epoch until the moment of abortion. Intuitively, the early stopping at the micro level discards the remaining data in the batch allocated for the current epoch. Therefore, it is necessary to exclude the discarded data from the loss calculation. Concretely, Equation (7) shows that the entropy value is divided by the total number of examples (N) in the epoch.

(7) $$J\left(w\right)=-\frac{1}{N}\sum_{i=1}^{N}\sum_{c=1}^{C}{1_{{y_{i}}_{\epsilon }C_{c}}}_{}\;\log_{p_{\mathrm{model}}}\left[y_{i}\epsilon C_{c}\right]$$

where C denotes the category (class) and $1_{{y_{i}}_{\epsilon }C_{c}}$ denotes the *i*th observation that belongs to the *c*th category. Such a calculation overlooks the effect of early stopping. To rectify such a drawback, our study introduces a gradual weighting coefficient δ into the loss function as shown in Equation (8). The calculation of δ value is shown in Equation (9).

(8) $$J\left(w\right)=-\frac{\delta }{N}\sum_{i=1}^{N}\sum_{c=1}^{C}{1_{{y_{i}}_{\epsilon }C_{c}}}_{}\;\log_{p_{\mathrm{model}}}\left[y_{i}\epsilon C_{c}\right]$$

(9) $$\delta =\frac{N}{l}$$

where l denotes the number of examples that were consumed so far during the current epoch. The coefficient δ reduces the weight of N according to the actual number of training examples that were used during the epoch. If an epoch stops early, the loss calculation is carried out based on the consumed data only. Therefore, the accuracy of loss estimation is improved, which consequently improves the accuracy of the model. The GCE is integrated into the detection model and used during the training phase to support the feed-forward and backpropagation.

### 2.5. Dataset Description

**Figure 2:**
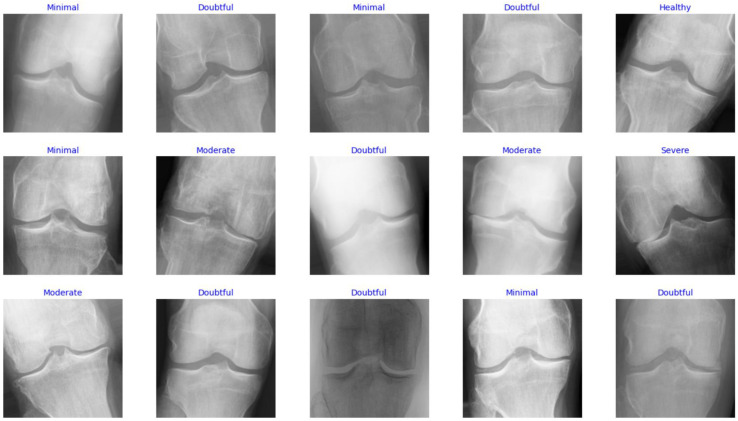
Samples of images in the dataset.

In this study, the Knee Osteoarthritis Severity Grading dataset is used to train and evaluate the performance of the proposed model. It contains knee X-ray images for OA detection and KL grading. Five gradings constitute the dataset labels as follows: healthy knee image (grade 0), doubtful joint space narrowing (JSN) with possible OA (grade 1 or healthy), confirmed OA and possible joint space narrowing (grade 2 or minimal), multiple moderate OA with confirmed JSN and mild sclerosis (grade 3 or moderate), and large OA with significant JSN and severe sclerosis (grade 4 or severe). The data are distributed based on the grades, such that there are 604 images for grade 0, 275 images for grade 1, 403 files for grade 2, 200 images for grade 3, and 44 images for grade 4. Figure 2 shows samples of images with various labels.

From the data distribution illustrated above, it can be seen that the dataset has a class imbalance, which might lead to classification bias toward the majority label. To mitigate this drawback, data augmentation was used to balance the training set so that each class contains 500 samples. Several techniques were employed to conduct the augmentation, including flipping, rotation, shifting, and zooming. Table 2 shows the augmentation parameter customization used in this study, which was determined experimentally.

The augmented samples were then added to the dataset and used for training the detection model. The dataset was divided into three subsets, training, validation, and testing, using the cross-validation method by which the data were sampled randomly, and the five labels were represented in all subsets. Table 3 shows the data distribution among the three subsets after conducting the K-fold cross-validation split.

### 2.6. Development and Evaluation Environment

This experiment was carried out in an MS Windows 10 machine with 16 GB RAM, 12th Gen Intel Core i 7, 4.7 GHz, and NVIDIA 1050 Ti GPU. Python with several libraries such as Tensor flow, Keras, Pandas, Numpy, Matplotlib, and Sci-kit learn was used to develop the DenseNet169 DL model.

### 2.7. Evaluation Metrics

This study makes use of the confusion matrix to evaluate the performance of the proposed model. On one side, the matrix shows the actual values, and on another side, it shows the predicted values. Then, the ratio of true positive, true negative, false positive, and false negative can be deduced. Several metrics were used to measure the performance of the model including accuracy, *F1* score, loss rate, precision, and recall. The following equations are used to calculate these metrics for multi-class classification:

(10) $$Accuracy=\frac{\sum TP+\sum TN}{\sum TP+\sum TN+\sum FP+\sum FN}$$

(11) $$Recall=\frac{\sum TP}{\sum TP+\sum FN}$$

(12) $$Precision=\frac{\sum TP}{\sum TP+\sum FP}$$

(13) $$F1\;Score=\frac{2\times Pr\times Re}{Pr+Re}$$

where TP, TN, FP, and FN represent the true positive, true negative, false positive, and false negative, respectively.

## 3. Results and Discussion

In this section, the experimental results are detailed. The experiments were conducted on the training dataset in three rounds. During the first round, we built a multi-class classifier using the five labels in the dataset. In the second round, another multi-class classifier was built using only three class labels. To achieve this, the class labels in the dataset were categorized into three classes, including healthy, moderate, and severe. Lastly, we built a binary classifier where the labels were put under two categories, healthy and unhealthy. The purpose of such multi-round training is to investigate the effect of multi-classes on the accuracy of the model. This helps to determine whether increasing the classes affects the ability of DenseNet169 to detect the stage of OA.

Table 4 summarizes the performance of the proposed model with respect to accuracy, F1 score, precision, and recall. The model applies the adaptive early stopping when the training process does not make any further improvements, which helps to prevent overfitting. The results show that the five-class classification achieved 0.62 accuracies, 0.65 F score, 0.58 precision, and 0.61 recall. For the three-class classification, the results show an increase in the accuracy to 0.93, F1 score to 0.90, precision to 0.91, and recall to 0.91. When we used the binary class classification, the results were increased to 0.94 for accuracy, F1 score, precision, and recall. The confusion matrix for the three classification tasks was used to calculate the performance metrics (accuracy, recall, precision and F1 score). The horizontal side of those matrices represents the actual labels, while the vertical side represents the predicted labels. The intersection between the actual and predicted labels determines the performance of the model as to whether it generates true positives (TP), true negatives (TN), false positives (FP), or false negatives (FN). Based on such a prediction, the accuracy of the model was calculated.

**Figure 3:**
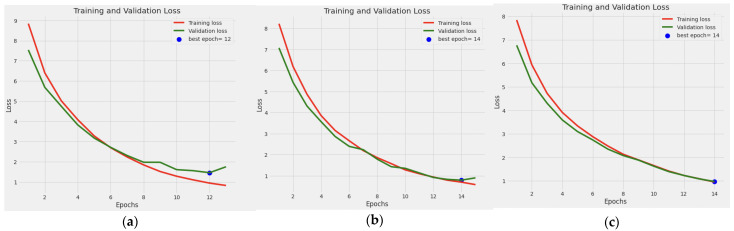
Training and validation performance of the model for the 5-class (**a**), 3-class (**b**), and 2-class (**c**) tasks over the training epochs.

Figure 3 shows the training and validation performance of the model for the three-class tasks (5-class (a), 3-class (b), and 2-class (c)) over the training epochs. It also shows the best fit where the training and validation curves intersect. It can be observed that the loss decreases in both the training and validation sets when the number of epochs increases. In the three classification tasks (i.e., five-class, three-class, and two-class classification), it can be noticed that the training loss was higher than the validation loss at the early epochs. While the training loss continues to decrease at the late epochs during the five-class training (3:a), the validation loss curve tends to flatten, which indicates that the loss does not improve by increasing the epochs. However, during the three-class and two-class classifications, both the training and validation losses overlap most of the time. The loss curves also show the effect of the adaptive early stopping technique as the training stops at 12 epochs (five-class), 13 epochs (three-class), and 14 epochs (two-class) when the model detects no more improvement on the validation set.

**Figure 4:**
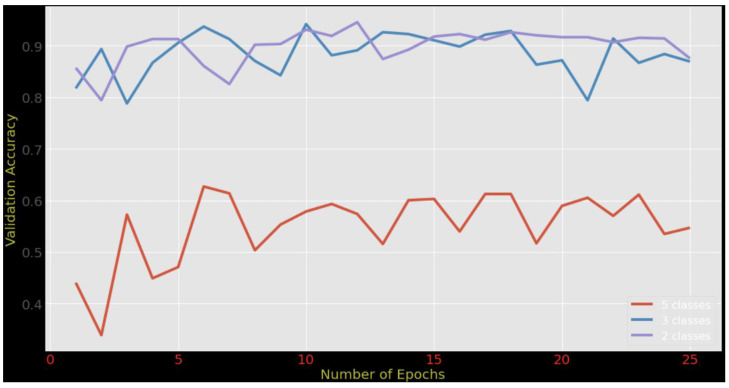
Adaptive DenseNet169 accuracy trending based on the number of epochs for 2-class, 3-class, and 5-class classification.

Figure 4 shows the comparison between five-class classification, three-class classification, and the binary classification that we conducted using our proposed model. The results were taken after each epoch using the validation set. It can be observed that the binary classification achieved the highest accuracy, while the accuracy of the five-class classification was the lowest. Moreover, it can be observed that the training accuracy increases when the number of epochs increases, until the number of epochs reaches 25, where we can see that the increase becomes less gradual. Furthermore, the comparison shows that the validation accuracy was not stable and oscillated around 0.6. This indicates that the data with five-class labels negatively affect the accuracy when new data are introduced to the model. The reason behind this drop in the model’s accuracy could be the overlapping between the class boundaries, which makes it difficult for the model to distinguish between the characteristics of those classes. The high training accuracy confirms such claim as the model overfits the training examples. In contrast, the validation accuracy for three-class classification and binary classification increased to around 0.9. This means that the model performed well when the target label was less granular but dropped when the labeling became more specific. This could be due to the inability of the model to explore discriminative features that represent the fine granular labels (the five-class case). One potential solution is to embed an attention layer as a feature selection mechanism into the model structure so that it can focus on a set of features relevant to the target classes.

**Figure 5:**
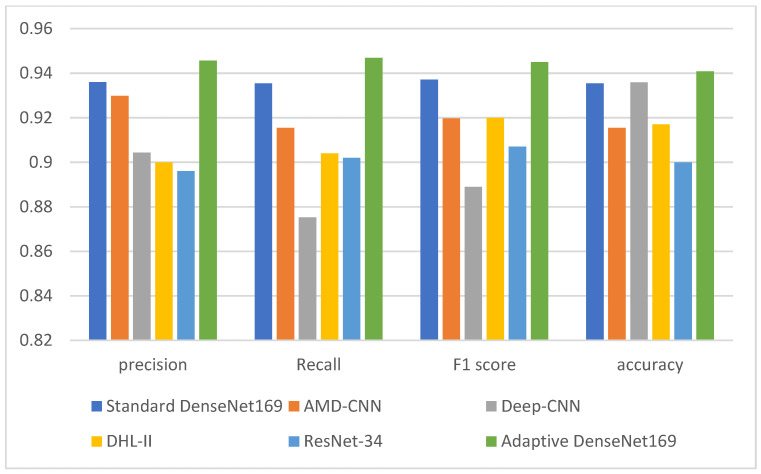
Comparison between the performance of the proposed model with the related models (Standard Densenet169, AMD-CNN [13], Deep-CNN [24], DHL-II [35], ResNet [29]) for the 2-class classification.

**Figure 6:**
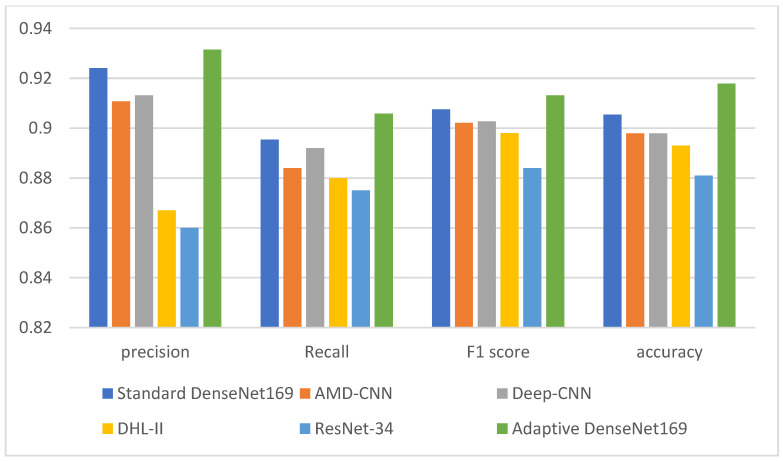
Comparison between the performance of the proposed model with the related models (Standard Densenet169, AMD-CNN [13], Deep-CNN [24], DHL-II [35], ResNet [29]) for the 3-class classification.

**Figure 7:**
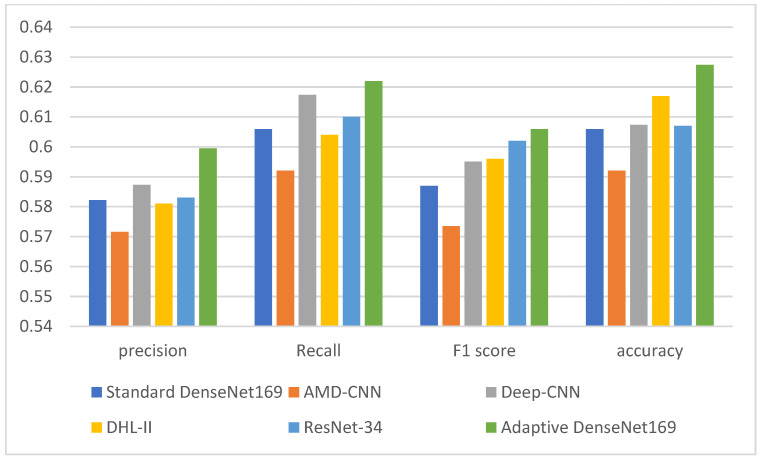
Comparison between the performance of the proposed model with the related models (Standard Densenet169, AMD-CNN [13], Deep-CNN [24], DHL-II [35], ResNet [29]) for the 5-class classification.

Table 5, Table 6 and Table 7 show a comparison between the proposed adaptive DenseNet169 model with the results of existing works. The comparison is also visualized in Figure 5, Figure 6 and Figure 7. The comparison was conducted between the proposed adaptive DenseNet169 and the standard DenseNet169. We also compared the performance of the proposed model with the existing studies related to pre-trained models for knee OA detection with early stopping capabilities, namely, AMD-CNN [13], deep CNN [24], DHL-II [35], and ResNet-34 [29]. In this comparison, we used several metrics, namely, precision, recall, F1 score, and accuracy. By comparing the results obtained from our model with those obtained by related works, it can be observed that the proposed model outperformed the previous models in terms of accuracy, recall, and precision. In the comparison, the proposed model as well as the models developed by existing works were trained using the same number of epochs. In our model, the adaptive early stopping was applied at a batch level, in which the model aborts the training of the respective epoch if new instances have little or no contributions to improving the validation accuracy.

The improved performance (in terms of precision, recall, F1 score, and accuracy) that our proposed adaptive DenseNet169 model shows over existing models in the two-class, three-class, and five-class classifications can be primarily credited to the incorporation of adaptive early stopping and the use of the gradual cross-entropy (GCE) loss estimation technique. Early stopping allows our model to run an adequate number of epochs with an appropriate batch size for each classification, preventing overfitting and avoiding premature termination of training when the epochs and batch sizes are underestimated. This adaptability allows for the dynamic readjustment of the patience parameter, which ensures optimal data utilization, consequently leading to maximized accuracy.

This improved performance is attributed to the efficacy of the GCE technique, which adaptively tunes the patience parameter based on the validation loss at the epoch level. Unlike conventional methods that solely depend on the number of epochs for setting the patience parameter, our model incorporates GCE to base the loss estimate on the data processed at the epoch level before early stopping. This technique negates the influence of discarded data on the loss calculation, ensuring more precise loss estimation. Hence, these combined strategies allow our model to adapt and learn more effectively and accurately, resulting in its improved performance over the existing models.

It can also be observed that the five-class classification achieved the lowest performance by all models across all metrics (precision, recall, F1 score, and accuracy). This can stem from the class imbalance, as the class “Healthy” has the highest number of images (2286), and the class “Severe” has the least number of images (173). This creates a significant discrepancy between the classes, and, as a result, the model might become biased toward the “Healthy” class, simply because it encounters more examples of this class during training, making it less capable of accurately detecting and differentiating between the less represented “Doubtful”, “Minimal”, “Moderate”, and “Severe” classes. To address this issue, multimodal deep learning can be an effective solution, as it leverages multiple types of data input, such as combining image data with structured clinical data. For example, the model could be trained on both X-ray images and corresponding clinical data such as patient age, weight, gender, pain levels, and other relevant health metrics. By integrating these additional data sources, the model could learn more complex representations and dependencies, leading to more accurate OA severity predictions. However, collecting and integrating diverse types of knee OA-related data (such as images, text, structured clinical data, etc.) can be challenging due to data privacy and protection regulations such as the Health Insurance Portability and Accountability Act (HIPPA). These regulations, either mandated by local or federal jurisdictions, strictly control the access to and usage of personal health information, thereby adding a layer of complexity to the data collection process for multimodal training. In addition, ensuring accurate alignment across different types of data is essential and non-trivial. For successful and accurate multimodal training, it is critical to ensure that all data types—the images, the textual information, and the structured clinical data—correctly correspond to the same entity, such as a patient. This alignment guarantees that the integrated data maintain their contextual relevance, thereby enabling the model to develop a coherent understanding of the information. Moreover, it can be difficult to understand which modality is contributing to the predictions and how they are interacting with each other. These challenges can be investigated further in future studies. Researchers could delve deeper into these issues, developing innovative solutions to streamline the alignment process across different data types, and enhance the interpretability of multimodal models.

## 4. Conclusions

In this study, we present a novel approach to improve the performance of DenseNet169-based knee osteoarthritis detection using X-ray images. Our approach utilizes an adaptive early stopping technique coupled with gradual cross-entropy loss estimation. We have shown that our approach improved the accuracy of knee osteoarthritis detection when compared to traditional early stopping techniques. Our results demonstrate that the proposed approach can lead to more accurate and efficient diagnostic tools for knee osteoarthritis. This study also investigates the effect of several types of classification on detection accuracy and shows that fewer classes generate accurate predictions. It is important to note that our approach is not without limitations. Further research is needed to investigate the generalizability of our method to other types of imaging modalities and to different types of osteoarthritis. Additionally, more efforts are needed to improve the model for multi-class classification when the number of classes increases. This is crucial for diagnosing the development of OA and identifying what stage the disease is at. The incorporation of other types of information, such as clinical data, may further improve the performance of the proposed method. Despite these limitations, our results are a promising step toward the development of more effective deep learning-based diagnostic tools for knee osteoarthritis.

## Figures and Tables

**Table 1:** DenseNet169 layered architecture.

Layer	Kernel Size	Parameters	Tensor Size
Convolution	Conv=7×7	Stride = 2, Relu	112×112
Pooling	MaxPool=3×3	Stride = 2	56×56
Dense1	Conv=1×1×6 Conv=3×3×6	Dropout = 0.2	56×56
Transition 1	Conv=1×1 AvgPool=2×2	Stride = 2	56×56 28×28
Dense 2	Conv=1×1×12 Conv=3×3×12	Dropout = 0.2	28×28
Transition 2	Conv=1×1 AvgPool=2×2	Stride = 2	28×28 14×14
Dense 3	Conv=1×1×32 Conv=3×3×32	Dropout = 0.2	14×14
Transition 2	Conv=1×1 AvgPool=2×2	Stride = 2	14×14 7×7
Dense 4	Conv=1×1×32 Conv=3×3×32	Dropout = 0.2	7×7
Classification	AvgPool=1×1 1000D (fully connected SoftMax)		1×1

**Table 2:** Augmentation optimization parameters.

Parameter	Value
horizontal_flip	True
rotation_range	25
width_shift_range	0.22
height_shift_range	0.23
zoom_range	0.25

**Table 3:** Data distribution among the training, validation, and testing subsets.

Subset Name	Number of Samples
Training set	2500
Validation set	826
Testing set	1656

**Table 4:** The performance of the proposed model with respect to the accuracy, F1 score, precision, and recall.

	Precision	Recall	F1 Score	Accuracy
2-class	0.9456	0.9469	0.9449	0.9408
3-class	0.9315	0.9058	0.9132	0.9179
5-class	0.5995	0.6220	0.6059	0.6274

**Table 5:** Comparison between the performance of the proposed model with the related models for the 2-class classification.

	Standard DenseNet169	[13]	[24]	DHL II [35]	ResNet [29]	Adaptive DenseNet169
Precision	0.936	0.9298	0.9043	0.9	0.896	**0.9456**
Recall	0.9354	0.9155	0.8753	0.904	0.902	**0.9469**
F1 score	0.9371	0.9197	0.8889	0.92	0.907	**0.9449**
Accuracy	0.9354	0.9155	0.9358	0.917	0.9	**0.9408**

**Table 6:** Comparison between the performance of the proposed model with the related models for 3-class classification.

	Standard DenseNet169	[13]	[24]	DHL II [35]	ResNet [29]	Adaptive DenseNet169
Precision	0.9241	0.9107	0.9132	0.867	0.86	**0.9315**
Recall	0.8954	0.884	0.892	0.88	0.875	**0.9058**
F1 score	0.9075	0.9021	0.9027	0.898	0.884	**0.9132**
Accuracy	0.9054	0.8979	0.8979	0.893	0.881	**0.9179**

**Table 7:** Comparison between the performance of the proposed model with the related models for the 5-class classification.

	Standard DenseNet169	[13]	[24]	DHL II [35]	ResNet [29]	Adaptive DenseNet169
Precision	0.5822	0.5716	0.5873	0.581	0.583	**0.5995**
Recall	0.6059	0.5921	0.6174	0.604	0.61	**0.622**
F1 score	0.587	0.5735	0.5951	0.596	0.602	**0.6059**
Accuracy	0.6059	0.5921	0.6074	0.617	0.607	**0.6274**

## Data Availability

Not applicable.
